# Successful Management in an Infant Patient of PHACE Syndrome with a Complicated Aortic Arch Anomaly

**DOI:** 10.1155/2022/5947951

**Published:** 2022-08-24

**Authors:** Shun Suzuki, Mitsuru Seki, Koichi Kataoka, Reina Koga, Tomoyuki Sato, Masaaki Kawada, Takanori Yamagata

**Affiliations:** ^1^Department of Pediatrics, Jichi Medical University, Tochigi, Japan; ^2^Pediatric Operating Suite and Intensive Care Unit, Jichi Medical University, Tochigi, Japan; ^3^Department of Pediatric Cardiology, Hiroshima City Hospital, Hiroshima, Japan; ^4^Department of Cardiac Surgery, Section of Pediatric and Congenital Cardiovascular Surgery, Jichi Medical University, Tochigi, Japan

## Abstract

PHACE syndrome is a congenital disorder often associated with a cervicofacial infantile hemangioma and complicated cardiovascular malformations. Patients with PHACE syndrome often have complex aortic arch anomalies, longer aortic stenosis or agenesis segments, and increased vascular tortuosity; therefore, perioperative management and surgical repair are challenging. We report a case of a female infant with PHACE syndrome and complex cardiovascular anomalies such as a double aortic arch associated with interruption of the left aortic arch, coarctation of the right aortic arch, patent ductus arteriosus, ventricular septal defect, and atrial septal defect. She was born at 36 weeks of gestation (birth weight, 2,150 g) and the diagnosis was confirmed by three-dimensional computed tomography. Because her patent ductus arteriosus did not close at first, her heart failure was managed preoperatively without prostaglandin *E*_1_. We initially attempted to promote weight gain. Surgical planning and simulation were performed using the patient-specific three-dimensional cardiovascular model created from computed tomography data. She underwent a successful aortic arch reconstruction by an end-to-side anastomosis with anterior patch augmentation at the age of 56 days. Detailed planning and simulation before surgery were vital in achieving favorable outcomes. Careful management and surgical planning using a patient-specific three-dimensional model are vital, especially in patients with complex malformations, such as in our case.

## 1. Introduction

PHACE syndrome (P: posterior fossa anomalies, H: hemangiomas, A: arterial lesions, C: cardiac abnormalities, E: abnormalities of the eye) (OMIM ^#^606519) is a congenital disorder that manifests as giant infantile hemangioma, arterial malformation of medium or large-sized arteries, cardiac abnormalities, and central nervous system anomalies [1]. An abnormal arterial malformation in patients with PHACE syndrome sometimes manifests as complex aortic arch anomalies, longer aortic stenosis or agenesis segments, and increased vascular tortuosity [2]. Therefore, managing the preoperative phase and the individual strategy for surgical repair is challenging for complex cardiovascular anomalies.

The effectiveness of surgical simulation using a three-dimensional (3D) model for complex cardiac malformations has been reported recently [3]. We report the successful preoperative and surgical management of an infant with a diagnosis of PHACE syndrome with a double aortic arch, left-sided interruption, and right-sided coarctation, via detailed image evaluation and a patient-specific 3D cardiovascular model.

## 2. Case Presentation

**Figure 1:**
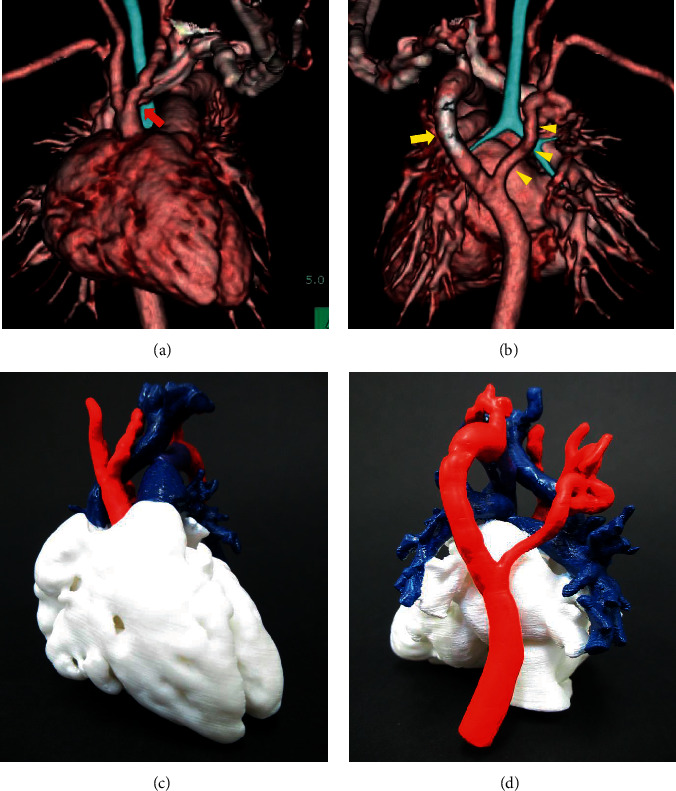
(a, b) Contrast-enhanced three-dimensional computed tomography images obtained at 8 days of age. (c, d) Patient-specific three-dimensional solid cardiovascular model based on computed tomography data at 8 days of age. Computed tomography scans were not electrocardiography-gated. (a) The frontal view. The left aortic arch was interrupted after the branching of the left common carotid artery (red arrow). (b) The rear view. This image shows the left distal arch connected to the ductus arteriosus (yellow arrow) and right distal arch (small yellow arrowheads). The right dorsal aorta was long and tortuous, with hemodynamic aortic coarctation. (c) The frontal view and (d) the rear view. Arteries are colored in red. Pulmonary arteries and veins are colored in blue. The patent ductus arteriosus is colored purple. The heart and vessel model was helpful to plan and simulate surgery.

A female infant was born at 36 weeks of gestation (birth weight, 2,150 g) at our institution. No cardiovascular abnormalities were diagnosed prenatally. She had a giant hemangioma from the right periorbital to the temporal region and a small hemangioma on the right shoulder at birth. Due to the finding of a systolic heart murmur one day after birth, echocardiography was performed, revealing a ventricular septal defect (VSD) and a secundum-type atrial septal defect (ASD). The VSD, 3.0 mm in diameter, was located in the membranous portion. On day 7, the patient showed poor suckling and tachycardia. Chest X-rays revealed a cardiothoracic ratio of 0.68 and pulmonary plethora, and electrocardiography suggested left ventricular hypertrophy. A detailed re-examination of the echocardiogram caused us to suspect interruption of the aortic arch (IAA) and patent ductus arteriosus (PDA). Eventually, contrast-enhanced computed tomography (CT) revealed a double aortic arch with left-sided IAA and right-sided coarctation of the aorta (CoA), PDA, and a retroaortic innominate vein (Figures 1(a) and 1(b)). CT also showed the absence of an internal carotid artery bifurcating from the common carotid artery and an ectopic thyroid gland, which could lead to hypothyroidism.

The patient showed an upper extremity percutaneous oxygen saturation of 90% without differential cyanosis and a blood pressure difference between the right upper and lower extremities of approximately 10 mmHg. Due to a left-to-right shunt caused by the PDA with a diameter of 5.0 mm, her heart failure progressed with an elevated brain natriuretic peptide level of 4,671 pg/ml. Treatment with diuretics and a phosphodiesterase 3 inhibitor (olprinone) attenuated the heart failure and her weight gain gradually improved. This complex combination of arch abnormalities and low body weight might have complicated surgical intervention in the early neonatal period; therefore, we decided to perform aortic arch reconstruction after weight gain. The PDA did not close at first, even without prostaglandin *E*_1_. Because the patient was at high risk of necrotizing enterocolitis, we carefully monitored the blood pressure in her lower extremities and her urine output and we used abdominal X-rays to evaluate her intestinal gas patterns. Although she exhibited no symptoms of necrotizing enterocolitis, echocardiography at 29 days showed a narrowing of the PDA to 3.5 mm in diameter. The systolic blood pressure difference between the right upper and lower extremities had increased to 40 mmHg. Because her heart failure did not progress, we continued medical therapy to help her gain weight.

We developed a patient-specific 3D solid model (Figures 1(c) and 1(d)) from multidetector CT data to plan and simulate the surgical intervention. We have previously reported the implementation of this method at our institution [4, 5]. Acrylonitrile-butadiene-styrene resin-based solid heart and vessel models were created using a commercially available personal 3D printer. In the heart model, we observed a large gap (approximately 2 cm) and a retroaortic innominate vein, which could potentially become an obstacle for anastomosis of the aortic arch between the ascending aorta and the left distal arch. Additionally, the right-sided aorta appeared to become narrow and tortuous. Therefore, reconstruction of the right aortic arch was considered unfavorable. Considering the complicated vascular malformations, the cardiovascular model helped us visualize the patient's condition and plan a feasible surgical approach.

**Figure 2:**
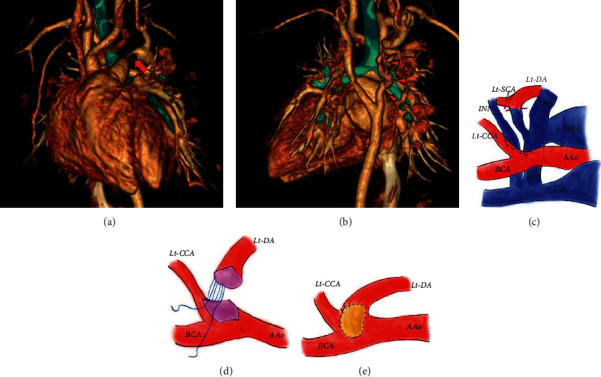
(a, b) Contrast-enhanced three-dimensional computed tomography images at 53 days of age. (c–e) Schema of the aortic reconstruction. (a) The frontal view. This image shows the narrowing of the patent ductus arteriosus (red arrow). (b) The rear view. Red small arrowheads indicate the right distal arch. The progression of narrowing and tortuousness of the right-sided aorta was shown compared to contrast-enhanced three-dimensional computed tomography images at 8 days of age. (c) The left aortic arch was interrupted, and the left subclavian artery arose from the left distal arch (interrupted aortic arch type B. Because there was a large gap, approximately 2 cm, between the ascending aorta and left distal arch, the left subclavian artery was resected from the distal arch to perform a direct anastomosis. The patent ductus ateriosus and adjacent ductal tissue were resected from the descending aorta. (d) The ascending aorta was cut back up to the undersurface of the proximal side of the left common carotid artery. The left distal arch was directly anastomosed to the undersurface of the arch (extended aortic arch anastomosis). (e) Anterior patch augmentation for the aortic arch was performed using glutaraldehyde-treated autologous pericardium. Superior vena cava (SCV), innominate vein (INN), ascending aorta (AAo), brachiocephalic artery (BCA), left common carotid artery (Lt-CCA), left subclavian artery (Lt-SCA), left distal arch (Lt-DA), main pulmonary artery (MPA), right pulmonary artery (RPA), left pulmonary artery (LPA).

**Figure 3:**
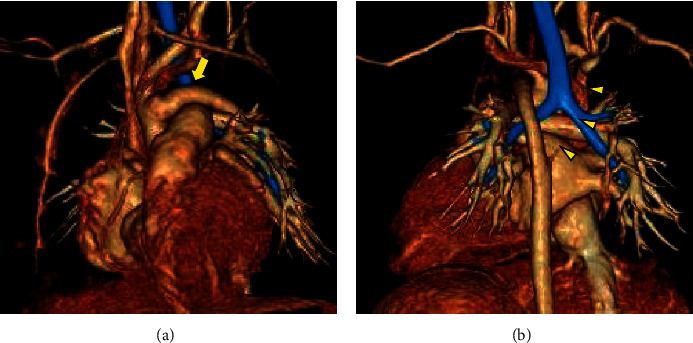
(a, b) Contrast-enhanced three-dimensional CT images obtained 21 days after surgery. (a) The front view and (b) the rear view. There is no stenosis in the reconstructed left aortic arch (yellow arrow) and the right distal arch is regressed (small yellow arrowheads).

While waiting for surgery, the PDA gradually became narrower, and an echocardiogram showed wall thickening of the left ventricle. Contrast-enhanced CT at 53 days of age (Figures 2(a) and 2(b)) revealed a progressive narrowing and tortuousness of the right aortic arch in addition to the PDA narrowing. Despite the gradual and progressive narrowing of the PDA, the diameter of the left dosal aorta was maintained. The use of prostaglandin *E*_1_ may be effective in certain cases; however, it was considered to fail in this case in terms of further weight gain owing to the progression of heart failure with increased pulmonary blood flow caused by PDA patency. The patient exhibited no pulmonary hypertension throughout her clinical course. At 56 days of age (body weight: 2.9 kg), she underwent aortic arch reconstruction and ASD closure as planned through the simulation. The cardiopulmonary bypass aortic cross-clamp times were 104 and 49 min, respectively. Isolated cerebral perfusion was performed for 38 min for cerebral protection. The aortic arch was reconstructed by end-to-side anastomosis distal to the left subclavian artery (Figures 2(c)–2(e)). Because of a large gap between the ascending aorta and left distal arch, the left subclavian artery was divided, and anterior patch augmentation was performed using glutaraldehyde-treated autologous pericardium. The right aortic arch and the closing perimembranous VSD were left untouched. At 3 weeks after surgery, contrast-enhanced CT showed no stenotic lesion of the repaired aortic arch or further regression of the right aortic arch (Figures 3(a) and 3(b)). The patient remained in the pediatric intensive care unit for 21 days after cardiac surgery and was discharged on the 34^th^ postoperative day. At two years of age (bodyweight: 10.3 kg), the small VSD was still patent without affecting the patient's hemodynamics, and her clinical course was good without recoarctation of the aorta or hypertension.

Chromosomal examination by G-banding and fluorescence in situ hybridization was negative for 22q11.2 deletion. Eventually, her condition was diagnosed as PHACE syndrome based on the findings of facial hemangiomas, cardiovascular malformations, and an ectopic thyroid gland, although brain magnetic resonance imaging showed no malformation of the posterior cranial fossa or cerebral vasculature. No ocular or sternal abnormalities were observed.

## 3. Discussion

PHACE syndrome is a congenital disorder that manifests as cervicofacial infantile hemangioma. Vascular anomalies in PHACE syndrome tend to manifest as tortuosity and long segments of aortic stenosis in unusual locations. Among infants with large facial hemangiomas, detailed examinations for cardiovascular and cerebrovascular anomalies are vital. Our case showed a complex combination of a double aortic arch, left-sided interruption, and right-sided coarctation, not previously reported. Although surgical management was challenging in this case, careful surgical planning using a patient-specific 3D model was useful.

PHACE syndrome is confirmed based on the presence of a hemangioma greater than 5 cm in the head, including the scalp, plus one major or two minor criteria, or a hemangioma in the neck or upper trunk, or proximal trunk/upper extremity plus two major criteria [6, 7]. Our case met the two major diagnostic criteria (anomalies of the major cervical arteries and an aortic arch anomaly) and three minor diagnostic criteria (a VSD, systemic venous anomalies (retroaortic innominate vein), and ectopic thyroid gland) for PHACE syndrome. Thyroid abnormalities should also be evaluated, in addition to detailed examinations of the brain, cerebral vessels, and eyes for complications of endocrine abnormalities [8]. In a prospective cohort study, 2.3% of children with infantile hemangioma met the criteria for PHACE syndrome [2]. Among infants with large facial hemangioma, 31% were diagnosed with this syndrome in one study [9]. In such patients, detailed examinations for cardiovascular anomalies should be performed in the neonatal period.

The differential diagnosis of the patient's cardiovascular anomalies included left aortic atresia; however, the absence of a cord between the left common carotid artery and the left subclavian artery excluded such a diagnosis. Hence, we considered two possibilities; one was “vascular ring, right aortic arch, left PDA, aberrant left subclavian artery, coarctation of the right arch;” and the other was “double aortic arch associated with left-sided IAA type B, right-sided coarctation of the dorsal aorta, left PDA.” The latter option was our final diagnosis as the right dorsal aorta of this patient was very tortuous and had regressed postoperatively, while the left dorsal aorta was as large as the aortic arch that provided blood flow from the pulmonary artery to the descending aorta. Moreover, the vessel diameter was preserved from the left aortic arch to the descending aorta after surgery.

For rare or complex cardiovascular anomalies, as in our case, perioperative management and surgical repair are challenging. Delicate or individualised preoperative management and decision-making for appropriate surgical intervention are crucial [10], and extensive aortic arch reconstruction with nonnative tissue is often required for complex lesions [11]. Our patient had a complex combination of a double aortic arch with left-sided IAA and right-sided coarctation and tortuosity of the aorta with no right internal carotid artery. We observed a large gap (approximately 2 cm) between the ascending aorta and left distal arch, as well as a retroaortic innominate vein, which might have impeded direct anastomosis of the aortic arch without residual obstruction. We also considered tracheal or esophageal compression owing to the vascular ring. CT scans revealed no evidence of such compression, and we expected that blood flow through the right aortic arch would contribute to maintaining the circulation of the lower body. Therefore, we determined that the right aortic arch did not need to be divided. In addition, there was a risk of compression owing to the circumflex aorta because the descending aorta was situated on the right. In hindsight, we realize that although no obvious compression was observed upon CT scanning, division of the right distal arch would have decreased the risk of compression.

Additionally, especially in low birth weight infants, aortic reconstruction carries a high risk of anastomotic and bronchial stenosis. Gaining as much weight as possible is more advantageous for aortic arch reconstruction repair without a nonnative tissue. In this regard, we considered the use of prostaglandin *E*_1_. Although administration of prostaglandin *E*_1_ is necessary for maintaining ductal patency in a neonate with an IAA, this might become a crucial factor in the high pulmonary blood flow-induced heart failure. Eventually, we decided against a continuous intravenous infusion of prostaglandin *E*_1_ considering the patient's poor weight gain due to heart failure. Indeed, this strategy requires careful follow-up; however, perioperative management similar to that in our case may become an option in cases with complex congenital cardiac anomalies.

The 3D model created from CT data helped us understand the complex vascular structure in this case. Vascular malformations vary among patients with PHACE syndrome, and careful and personalised surgical planning is essential. In such cases, the use of 3D models may be especially useful. Patient-specific 3D models are useful for planning and simulating surgery to treat complex congenital cardiovascular diseases [3]. Recent improvements in 3D printing technology have made it possible to rapidly and easily generate cardiovascular models using commercially available personal 3D printers. Although CT images are adequate for surgical planning, it is difficult to understand the actual size of the patient's heart only from CT images. Confirming the actual size by palpation before surgery can be advantageous, especially in cardiac surgery for pediatric patients. This can also help increase the success probability of the procedure and shorten operation time, thus leading to favorable patient outcomes.

In conclusion, a detailed investigation of PHACE syndrome should be performed in patients with large cervicofacial hemangioma. Surgery for complex congenital heart diseases is challenging. Especially in PHACE syndrome, in which vascular anomalies are highly individualized, careful surgical planning with detailed imaging evaluation for each case is vital. A patient-specific 3D cardiovascular model can be beneficial for surgical simulation. Future improvements in technology will allow the widespread use of 3D cardiovascular models in clinical practice, improving patient outcomes.

## Data Availability

Owing to the nature of this research, the patient's guardians did not agree for their data to be shared publicly, and no supporting data are available.

## Abbreviations

ASD: Atrial septal defect
IAA: Interruption of the aortic arch
PDA: Patent ductus arteriosus
CT: Computed tomography.
CoA: Coarctation of the aorta.
